# ImitateCholec: A Multimodal Dataset for Long-Horizon Imitation Learning in Robotic Cholecystectomy

**DOI:** 10.1038/s41597-025-06526-z

**Published:** 2026-01-16

**Authors:** Pascal Hansen, Ji Woong Brian Kim, Antony Goldenberg, Juo Tung Chen, Yuanzhe Amos Li, Anton Deguet, Brandon White, De Ru Tsai, Richard Cha, Jeffrey Jopling, Paul Maria Scheikl, Axel Krieger

**Affiliations:** 1 Laboratory for Computational Sensing and Robotics, Johns Hopkins University, Baltimore, 21218 USA; 2 Division of Intelligent Medical Systems, German Cancer Research Center, Heidelberg, Germany; 3 Department of Computer Science, Stanford University, Stanford, 94305 USA; 4 Department of Surgery, Johns Hopkins University, Baltimore, 21218 USA; 5 Optosurgical, Columbia, 21046 USA; 6 Amazon Robotics, Berlin, Germany

**Keywords:** Biomedical engineering, Endoscopy, Computer science

## Abstract

The growing global shortage of skilled surgeons underscores the need for intelligent, assistive technologies in the operating room. To address this challenge, we introduce ImitateCholec, a publicly available dataset specifically designed to advance autonomous robotic systems during the critical clipping and cutting phase of laparoscopic cholecystectomy. The dataset comprises over 18,000 demonstrations from 34 *ex vivo* porcine cholecystectomies, totaling approximately 20 hours of data. Each clipping and cutting phase recorded in the dataset is segmented into 17 distinct surgical tasks. ImitateCholec uniquely integrates endoscopic videos captured from multiple camera perspectives with comprehensive kinematic data acquired through the da Vinci Research Kit. Both optimal demonstration executions and recovery maneuvers were systematically recorded, enabling the training of imitation learning models capable of robustly addressing real-world surgical variability. Primarily, ImitateCholec facilitates imitation learning for long-horizon surgical workflow execution, significantly advancing the development of autonomous robotic systems toward achieving phase-level autonomy and, ultimately, full procedural autonomy. Additional supported applications include surgical workflow modeling, error recognition, and surgical tool pose estimation.

## Background & Summary

Minimally invasive surgery has significantly improved patient outcomes by reducing postoperative recovery times and complications compared to traditional open surgical procedures^1^. Techniques such as laparoscopic surgery enable surgeons to perform operations through small incisions, employing specialized instruments to access internal organs with minimal tissue damage. Advances beyond traditional laparoscopy have led to the development of robot-assisted surgery, significantly enhancing surgical precision, dexterity, and accessibility to anatomically challenging regions^2^. A prominent example is laparoscopic and robotic cholecystectomy—the surgical removal of the gallbladder—which is routinely performed worldwide, accounting for over 750,000 procedures annually in the United States alone^1^.

Despite these technological advances, the healthcare sector continues to face a critical shortage of surgical personnel. The increasing demand for surgical procedures, driven by an aging population and broader access to healthcare, has significantly strained existing medical infrastructure. Studies predict a shortage of approximately 20,000 general surgeons in the United States by 2030^3^. This shortage exacerbates the burden on surgical teams, resulting in extended working hours, heightened stress levels, surgeon burnout, and potentially compromised patient care quality^4^.

To mitigate these challenges, computer-assisted surgical systems leveraging existing robotic surgical infrastructure have emerged as promising solutions to address the shortage of skilled surgical personnel^5^. Assistive technologies, such as automated surgical workflow analysis, can streamline clinical documentation and significantly reduce postoperative workload^6^. Early autonomous surgical robotic systems have already demonstrated their capability to perform complex, short-duration tasks with high precision under controlled conditions^7^, highlighting their potential to alleviate surgical workloads by automating longer-horizon workflows such as individual surgical phases and, in the longer term, contributing to more comprehensive procedural support.

Open-source datasets such as Cholec80^8^, CholecT50^9^, and HeiCo^10^ have substantially advanced surgical workflow analysis by providing labeled surgical phases and actions for laparoscopic procedures. However, these datasets are restricted to laparoscopic video data, lacking essential kinematic information required to develop autonomous robotic surgical policies. Other datasets, including JIGSAWS^11^, designed primarily for gesture recognition in simplified tabletop settings, and CRCD^12^, focused on robotic clutch and camera operations, contain visual and kinematic data but are limited in task variability and scale. These limitations restrict their effectiveness in training autonomous surgical systems capable of generalizing to complex, realistic surgical scenarios. Developing autonomous systems for long-horizon tasks via imitation learning demands extensive datasets with numerous diverse task executions^13^. While extensive datasets supporting complex, long-horizon imitation learning exist in general robotics^14,15^, such comprehensive datasets are notably absent in surgical robotics, representing a critical barrier to progress in autonomous surgery.

**Fig. 1:**
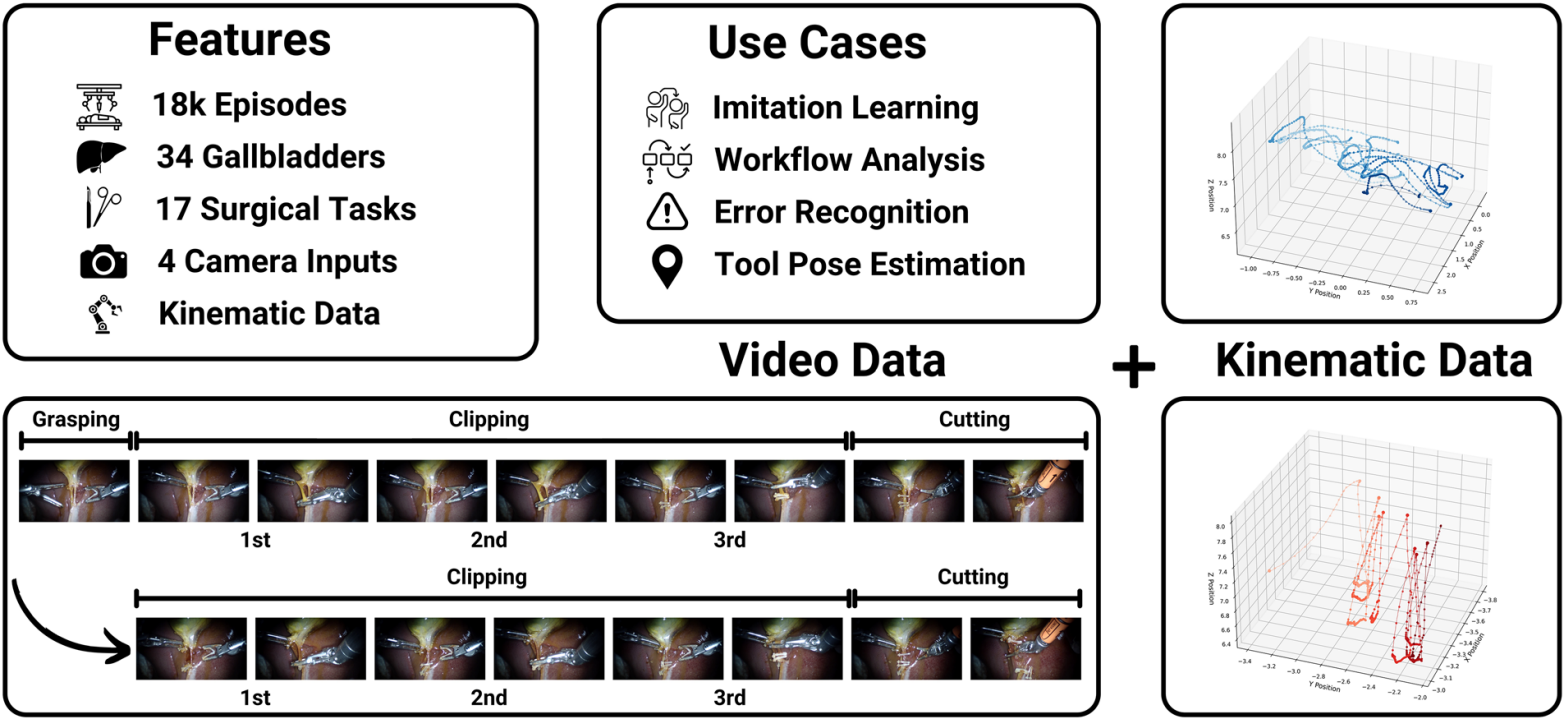
Overview of the ImitateCholec Dataset. The multi-modal ImitateCholec dataset comprises over 18,000 demonstrations across 17 surgical tasks (≈ 20 hours of data), integrating endoscopic video and instrument kinematics. The dataset is designed for imitation learning of long-horizon surgical workflows and uniquely includes both ideal executions and annotated recovery maneuvers for robust error detection and correction. Further use cases comprise surgical workflow analysis and surgical tool pose estimation.

To overcome these limitations, this paper introduces both a novel robotic surgical dataset and an associated structured data acquisition approach explicitly designed for imitation learning of long-horizon surgical phases, forming the basis for training SRT-H^16^, an autonomous framework for executing such surgical phases fully autonomously. Our systematic data acquisition includes extensive visual and kinematic recordings from 34 ex vivo porcine cholecystectomies, comprising over 18,000 surgical task demonstrations, specifically targeting the critical clipping and cutting phase across 17 distinct surgical tasks (see Fig. 1). Crucially, our dataset captures not only optimal task executions but also corrective maneuvers, enabling imitation-learning policies to robustly handle real-world surgical variability and recovery from errors. This comprehensive dataset and data acquisition approach provide an essential foundation for developing next-generation policies that can autonomously execute surgical workflows at the phase level and, eventually, achieve procedure-level autonomy. Using this data collection approach, also other related surgical multi-step long-horizon workflows could be recorded such as suturing or stapling. Moreover, the integration of kinematic information provides an opportunity to analyze the impact of multi-modal inputs on model robustness and reliability, *e.g*. in surgical workflow analysis, particularly in scenarios involving visual occlusions common in surgical environments. Finally, the dataset’s design naturally supports research in surgical tool pose estimation, further broadening its applicability and potential impact in surgical robotics research.

## Methods

### Data Acquisition

The following outlines the data acquisition process used at Johns Hopkins University for collecting visual and kinematic data during the clipping and cutting phases of *ex vivo* porcine cholecystectomy. All gallbladder organs were sourced from Animal Technologies, Inc. (Tyler, TX, USA). The data acquisition description is structured into four parts detailing the applied hardware and software setup, the experimental protocol followed, and the characteristics of both the recorded vision and kinematic data.

#### Setup

For the data acquisition, we utilized a da Vinci Research Kit (dVRK) Si system^17^. A dVRK Si has three robotic arms for manipulation tasks (referred to as patient side manipulators (PSMs): PSM1, PSM2, PSM3) and one robotic arm for holding the endoscopic camera (referred to as endoscope camera manipulator (ECM)).

A 12 mm, 0-degree stereo endoscope (Intuitive part number: 380609) is used for collecting video data capturing the surgical target area. Wrist cameras were mounted on the surgical instruments, 3.5 cm from the base of the instrument shafts, to improve the depth perception for the autonomous robot policy, as shown in^7^.

**Fig. 2:**
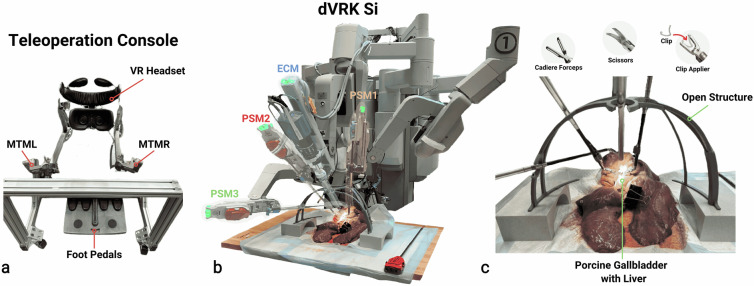
Surgical Robot Setup. **(a)** The teleoperation console, which consists of the MTMs, MTML and MTMR, the VR headset for 3D vision of the da Vinci Si endoscope input and the foot pedals for camera and clutch control, and another pedal to start the optimal and recovery demonstration recordings. **(b)** The dVRK Si surgical robot, consisting of three surgical arms the PSMs, PSM1-PSM3, using Cadiere forceps on PSM2 and PSM3 for grasping of the gallbladder, and PSM1 with a medium clip applier for clipping and a curved scissors for cutting. On the ECM a 12 mm, 0-degree stereo endoscope is attached. **(c)** The placement of the *ex vivo* tissue is enlarged together with the open structure. On the top, the used instruments are shown. Note that this image shows the *ex vivo* tissue in a relaxed position before setting it into a vertical position with PSM3. Below the ex-tissue is a rack that supports the tissue and prevents it from tearing when it is later placed into a vertical position.

The clipping and cutting are performed by PSM1 with a medium clip applier (Intuitive part number: 420327) and curved scissors (Intuitive part number: 420178). PSM2, equipped with a Cadiere forceps (Intuitive part number: 420049), is used to grasp the neck of the gallbladder and manipulate it to better separate and expose the tubes. In order to replicate the positioning of the key anatomical areas (gallbladder, cystic duct, and cystic artery) from human cholecystectomy procedures, the Cadière forceps on PSM3 holds the *ex vivo* tissue statically in an almost vertical position during the entire procedure. Once the optimal positioning for the data acquisition is found, PSM3 and ECM are kept stationary in their positions. Additionally, a computer-aided design computer-aided design (CAD) modeled custom rack is placed below the *ex vivo* tissue to further hold it in place and prevent tearing of the tissue. For standardized port locations of the surgical arms, a cad modeled open structure, based on port locations of a human cholecystectomy, is positioned in between the *ex vivo* tissue and the surgical robot. The open design specifically facilitates frequent clip reloading and tool switching. Throughout the procedure, a clamp has been applied on the end of the cystic duct to prevent bile (fluid stored in the gallbladder) from flowing out of the opening of the cystic duct. The surgical setup is demonstrated in Fig. 2.

**Fig. 3:**
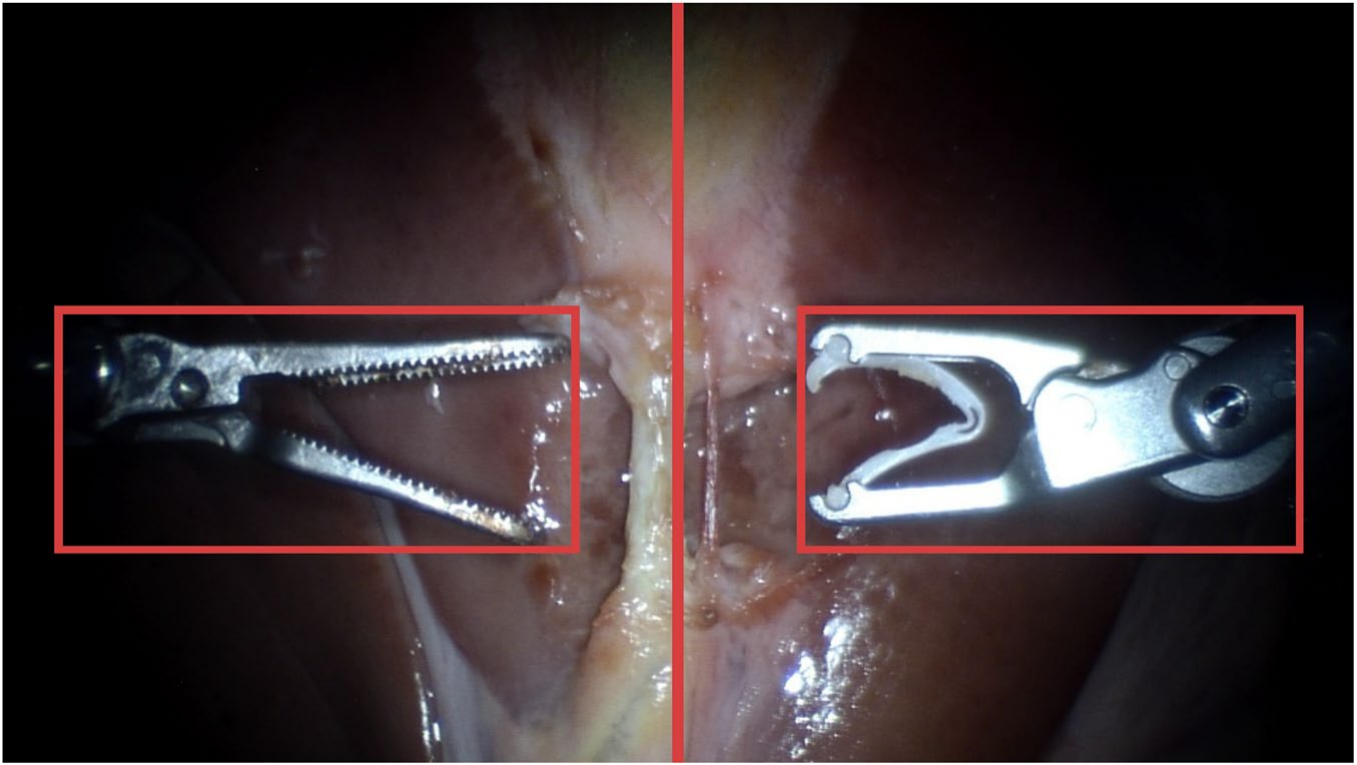
PSM1 & PSM2 Home Positions. The home positions for both instruments are displayed, which are added to the view of the data collector within the VR headset for a standardized data acquisition.

During the data acquisition, a data collector is sitting on the teleoperation console, which is displayed in Fig. 2A. Through the virtual reality (VR) headset connected to the teleoperation console, the data collector has a 3D vision of the surgical scene from the da Vinci Si stereo endoscope, similar to the view accessible in the original da Vinci teleoperation console. In the VR headset, we projected a center line (indicating the desired position for the tubes) and two bounding boxes (for PSM1 and PSM2) within the view of the data collector, see Fig. 3, to facilitate the standardized positioning of two instruments at the start and end of certain recordings. These standardized positions are later referred to as the home positions for PSM1 and PSM2.

The master tool manipulators (MTMs), MTML and MTMR, are used to control the two selected instruments, here the instruments on PSM1 and PSM2. The foot pedals, here from a da Vinci Classic / S foot pedal tray, can be used to readjust the camera and activate the clutch. The remaining foot pedals are used to control the starting and stopping of different types of recordings without moving out of the teleoperation console. During the data acquisition, all data streams were concurrently recorded at 30 Hz via robot operating system (ROS)^18^ using a publisher-subscriber pattern, *i.e*., having for each stream a separate ROS topic.

#### Protocol

**Fig. 4:**
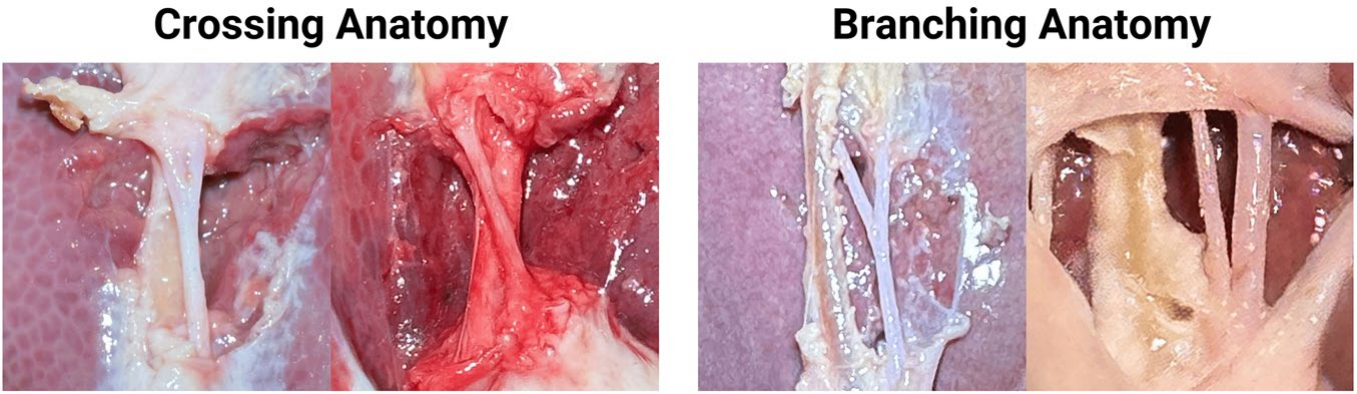
Examples of Excluded Anomalous Anatomy. The images show examples of *ex vivo* tissues that were excluded due to anomalous anatomy. This includes cases where the cystic duct and artery cross each other, as well as cases where the cystic artery exhibits branching.

The *ex vivo* porcine tissues used in this study consist of undissected gallbladders that remain attached to the liver. Prior to the clipping and cutting phase, the tissues must undergo dissection. The Calot’s triangle dissection is performed by blunt dissection using Maryland forceps (Intuitive part number: 420172) to achieve the critical view of safety (CVS), ensuring clear visibility of both the cystic duct and artery for safe clipping and cutting. When *ex vivo* tissues showed abnormal anatomical structures, such as a cystic artery crossing the cystic duct or branching anomalies (see Fig. 4), these tissues were excluded from the study. Thus, only tissues with two separable tubes were selected. Approximately 10% of tissues have been excluded based on such anatomical abnormalities.

**Fig. 5:**
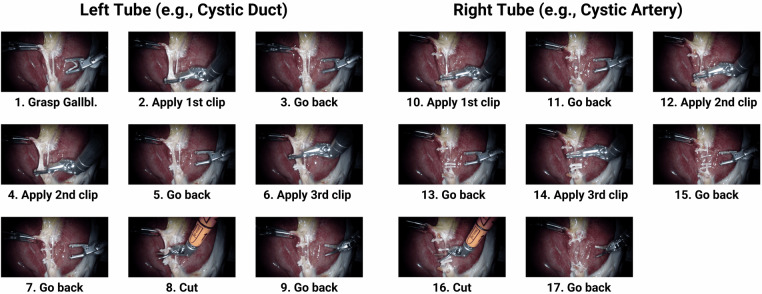
Clipping & Cutting Phase Workflow. Visualization of the 17 surgical tasks. From *grasping* the gallbladder to *clipping* and *cutting* the left tube (typically the cystic duct) as well as the right tube (typically the cystic artery). After each clipping or cutting task follows a *going back* to the home position task for reloading a clip or exchanging the instrument.

Once the gallbladders were dissected and the CVS achieved, data acquisition was performed by two experienced, non-clinical research assistants, each trained by a surgical resident with extensive expertise in performing cholecystectomies. The clipping and cutting phase was segmented into 17 more fine-grained surgical tasks, based on the terminology established in the 2021 SAGES consensus^19^, as illustrated in Fig. 5. First, the left tube (typically the cystic duct) is targeted, and after the cut, the focus shifts to the right tube (typically the cystic artery). The initial surgical task starts with both instruments in their standardized home positions and consists of *grasping the gallbladder* neck with the Cadière forceps while retracting the gallbladder neck. In task 2, the *first clip* is placed on the bottom of the *left tube*, followed by task 3, where the clip applier *goes back to the home position* to reload a new clip. At the end of the *going back* task, the clip applier is at the home position, and the gallbladder neck is relaxed by releasing the retraction to prevent tissue damage. Tasks 4 and 6 involve *clipping the second and third clips* for the *left tube*, with the second clip placed close to the first clip and the third clip placed at the top of the left tube. From task 4 onward, all *clipping and cutting* tasks include the retraction of the gallbladder neck. Tasks 5 and 7 are the *going back* tasks after the *second and third clip*, respectively, and are identical in their execution to task 3. After the three clips on the left tube are set, the *cutting* of the *left tube* is performed in task 8, followed by *going back to* the PSM1 *home position* in task 9. The clipping and cutting are then repeated for the *right tube*.

In addition to the optimally executed surgical tasks described above, also alternative versions of all grasping, clipping, and cutting tasks were recorded. These alternative versions begin with atypical instrument placements to simulate scenarios in which an error has occurred that needs to be corrected. The alternative executions are referred to as *recovery* demonstrations, while the standard executions are referred to as *optimal* demonstrations.

In order to maximize the number of demonstrations per *ex vivo* tissue and retain the condition of the tissue, specific techniques have been employed. Each surgical task has been recorded multiple times on the same tissue before moving on to the next task. For clipping, clips with a disabled latching mechanism were used, allowing repetitive clipping motions without fully locking onto the duct or artery. For cutting motions, the scissors were positioned around the tubes but not fully closed to keep the tubes in the same condition for the subsequent demonstrations. Only in the final demonstrations of the clipping and cutting, which are recorded continuously with the subsequent *going back* task, an unmodified clip was used or the cut performed. To prevent tissue degradation during the data acquisition, which can take multiple hours, the tissues were kept moist during the experiments.

#### Vision Data

The frames of the da Vinci Si stereo endoscopic video were saved, for both the left and the right image, with a downsampled resolution of 960 × 540 from the original resolution of 1920 × 1080. This reduces storage demands while retaining the level of detail required by modern computer vision models. The wrist camera frames, from PSM1 and PSM2, were recorded at a resolution of 640 × 480. Both the da Vinci and wrist camera frames were saved as JPG files.

#### Kinematic Data

**Fig. 6:**
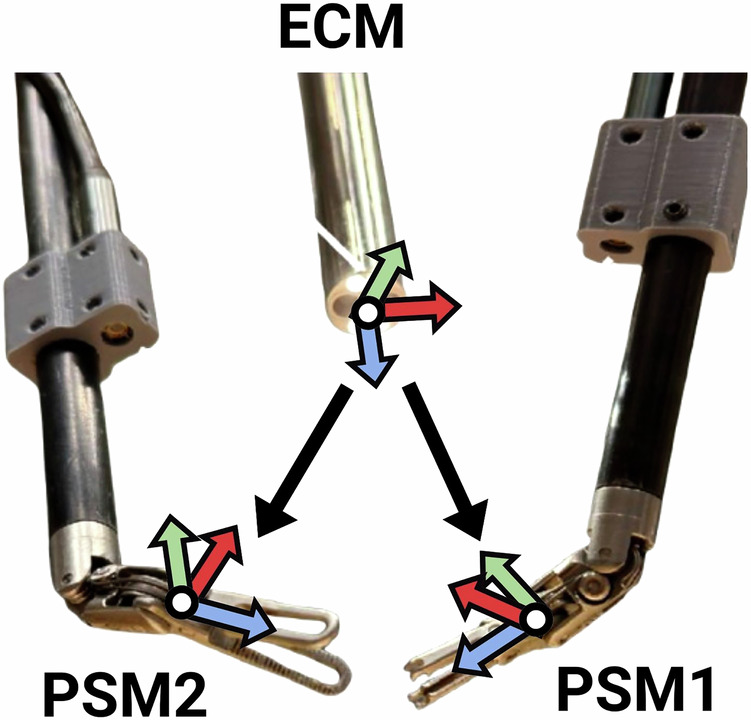
PSM Poses. Illustration of the PSM1 and PSM2 poses in the camera (ECM) reference frame.

The kinematic data collected from the da Vinci robotic system includes measurements from the two active PSMs (PSM1 and PSM2), the stationary positioning of PSM3, and the ECM. For each manipulator, the Cartesian pose of the instrument tip is recorded as a 7-dimensional vector, including 3D translation coordinates **(x,y,z)** in meters and a quaternion **(x,y,z,w)** representing orientation. The ECM tip serves as the reference frame for this setup, with the kinematic data from each PSM measured *w.r.t*. the ECM frame, as illustrated in Fig. 6. The local instrument tip Cartesian pose represents the pose *w.r.t*. the remote center motion (RCM) position of its arm, which was measured for the ECM and all PSMs.

In addition to capturing the Cartesian poses, the joint states of each arm—which represent the rotational and translational configurations of the manipulator’s joints—were recorded to comprehensively characterize the kinematic state of the da Vinci Si surgical system. The ECM comprises four joints, denoted as ${\{{\theta }_{i}\}}_{i=1}^{4}$, with joints 1-2 being rotational (in radians), joint 3 being translational (in millimeters), and joint 4 being rotational. Each of the PSMs include six joints, denoted as ${\{{\theta }_{i}\}}_{i=1}^{6}$, with rotational joints expressed in radians and the prismatic joint (joint 3) expressed in millimeters. For each PSM, the jaw opening angle *θ* is also provided, expressed in radians.

Besides the end-effector tip positions of the surgical instruments, kinematics of the set-up joints (SUJs) for the ECM and all PSMs are included. The SUJs kinematics are given for their base Cartesian poses, encoded as 7-dimensional vectors comprising 3D translation components (x, y, z) in meters and orientation in the form of a quaternion (x, y, z, w). Furthermore, the SUJs joint configurations are recorded as 4-dimensional vectors of joint values ${\{{\theta }_{i}\}}_{i=1}^{4}$, with the first joint being translational and expressed in millimeters, and the remaining joints being rotational and expressed in radians.

### Data Curation

All demonstrations were systematically reviewed to ensure correctness and completeness across all recorded modalities, including video and kinematic data. Recordings were temporally trimmed to exclude non-informative segments at the beginning or end, thereby preserving only the relevant portions of each demonstration. Since the actual tissue cutting occurs exclusively in the final repetition of each cutting task, the corresponding kinematic trajectories were augmented by linearly interpolating the jaw opening value toward a fully closed state, thereby simulating the instrument’s closing motion. Manual labeling was not required, as each surgical task was recorded independently. Consequently, demonstrations within each *ex vivo* tissue sample are organized by surgical task, enabling direct use of folder names as task labels where needed.

## Data Records

**Fig. 7:**
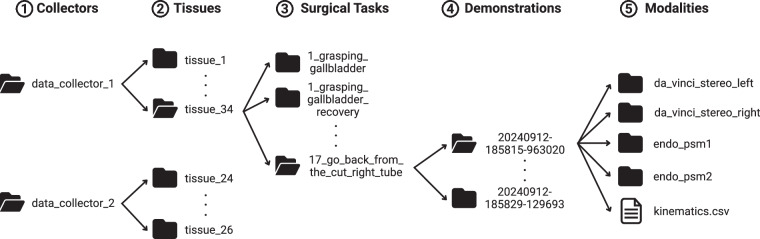
Dataset Folder Structure Overview. The folder structure of the ImitateCholec dataset consists of five levels: (1) collectors, (2) tissues, (3) surgical tasks, (4) demonstrations, and (5) modalities.

The dataset is hosted on the Johns Hopkins Research Data Repository^20^. The repository is publicly accessible without user registration. The data is organized in a five-level directory hierarchy, as illustrated in Fig. 7: Level 1 folders correspond to individual data collectors. Within each collector folder (Level 2), there is one subfolder per *ex vivo* tissue sample. Each tissue folder (Level 3) contains subfolders for each of the 17 surgical tasks. Only tissues 1, 11, 12, and 16 are missing one or more task recordings; all others include the full set of 17 optimal tasks. When recovery demonstrations were recorded, they were saved in a new surgical task folder named after the surgical task with the suffix _recovery. Within each task folder (Level 4), each demonstration is stored in a separate folder named according to its recording start timestamp. The number of demonstrations per task varies by tissue and task. Each demonstration folder (Level 5) contains: A kinematic.csv file. Four image subfolders: da_vinci_stereo_left, da_vinci_stereo_right, endo_psm1, and endo_psm2. Images are saved as JPG files named frame_<frame_id>.jpg, with <frame_id> running from 0 to the last frame index. Table 1 provides a detailed description of each column in kinematic.csv.

**Table 1:** Feature Descriptions for the da Vinci Robot System. Description of the features, their dimensions, and units in the da Vinci robot system. For all features with an asterisk *, there exist current values and target values. The same features were recorded for PSM3, which was kept stationary during the demonstrations.

Type	Features	Dim	Description (Units)
ECM	Endoscope Tip Cartesian Pose	7	Translation {*x*, *y*, *z*} (m), Quaternion {*x*, *y*, *z*, *w*}
	Local Instrument Tip Cartesian Pose	7	Translation {*x*, *y*, *z*} (m), Quaternion {*x*, *y*, *z*, *w*}
	Arm Joint State*	4	Translation: *θ*_3_ (mm), Rotation: *θ*_1,2,4_ (rad)
**PSM1**	Instrument Tip Cartesian Pose*	7	Translation {*x*, *y*, *z*} (m), Quaternion {*x*, *y*, *z*, *w*}
	Local Instrument Tip Cartesian Pose	7	Translation {*x*, *y*, *z*} (m), Quaternion {*x*, *y*, *z*, *w*}
	Arm Joint State*	6	Translation: *θ*_3_ (mm), Rotation: *θ*_1,2,4−6_ (rad)
	Instrument Jaw Joint State*	1	Rotation: *θ* (rad)
**PSM2**	Instrument Tip Cartesian Pose*	7	Translation {*x*, *y*, *z*} (m), Quaternion {*x*, *y*, *z*, *w*}
	Local Instrument Tip Cartesian Pose	7	Translation {*x*, *y*, *z*} (m), Quaternion {*x*, *y*, *z*, *w*}
	Arm Joint State*	6	Translation: *θ*_3_ (mm), Rotation: *θ*_1,2,4−6_ (rad)
	Instrument Jaw Joint State*	1	Rotation: *θ* (rad)
**SUJ-ECM**	Base Cartesian Pose	7	Translation {*x*, *y*, *z*} (m), Quaternion {*x*, *y*, *z*, *w*}
	Arm Joint State	4	Translation: *θ*_1_ (mm), Rotation: *θ*_2−4_ (rad)
**SUJ-PSM1**	Base Cartesian Pose	7	Translation {*x*, *y*, *z*} (m), Quaternion {*x*, *y*, *z*, *w*}
	Arm Joint State	4	Translation: *θ*_1_ (mm), Rotation: *θ*_2−4_ (rad)
**SUJ-PSM2**	Base Cartesian Pose	7	Translation {*x*, *y*, *z*} (m), Quaternion {*x*, *y*, *z*, *w*}
	Arm Joint State	4	Translation: *θ*_1_ (mm), Rotation: *θ*_2−4_ (rad)

**Fig. 8:**
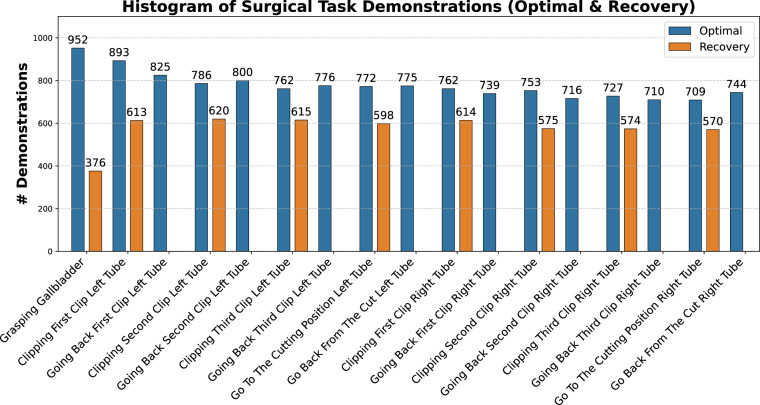
Number of Demonstrations per Surgical Task. Number of demonstrations recorded for each of the 17 surgical tasks, for the *optimal* and *recovery* demonstrations of data collector 1 and data collector 2.

The resulting dataset consists of a total of 34 *ex vivo* tissues. The total number of demonstrations is around 13,188 optimal and about 5,155 recovery demonstrations, which are about 20 hours of data. A dataset summary can be found in Table 2 and more details on the demonstration distribution are shown in Fig. 8.

**Table 2:** Dataset Summary. Statistics for the data collected by the two data collectors.

		Data Collector 1	Data Collector 2
**Num. Porcine Tissues**		31	3
**Optimal Demos**	Num.	12,303	885
	Images	1,457,249	126,325
	Time (s)	48,575	4,211
**Recovery Demos**	Num.	4,892	263
	Images	691,425	40,297
	Time (s)	23,048	1,343

## Technical Validation

### Validation of Vision Data

**Fig. 9:**
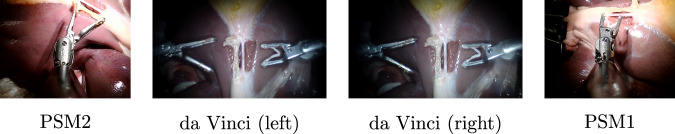
Camera Input. Images from the wrist cameras (PSM2 and PSM1) with the left image from the da Vinci Si stereo endoscope.

To ensure dataset reliability and quality, we implemented comprehensive validation procedures focusing initially on visual data. Each demonstration was carefully reviewed to confirm it depicted a complete and correctly executed task within the defined task boundaries (see Fig. 9). Specifically, the first and last frames of each video were examined to confirm proper initiation and completion, ensuring that instruments started in the designated start pose and successfully completed the intended task. Additionally, each recording was checked to confirm it contained no segments from previous or subsequent tasks, maintaining strict adherence to the predefined task boundaries.

### Validation of Kinematic Data

**Fig. 10:**
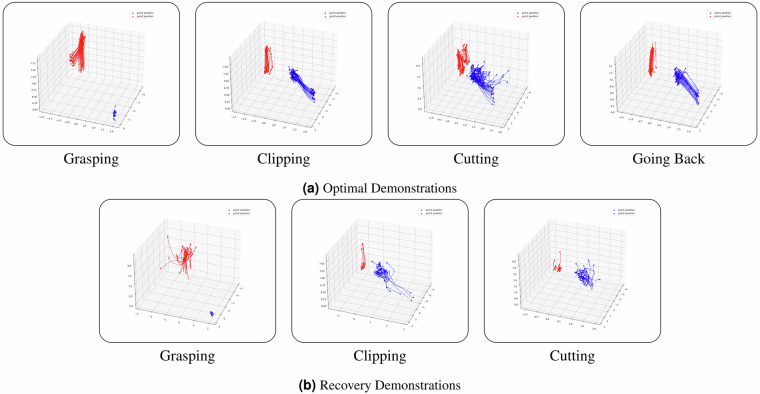
Kinematics (Optimal and Recovery Demonstrations). Examples of *optimal* and *recovery* demonstration kinematics recorded for the main tasks, *i.e*., *grasping*, *clipping*, *cutting* (with *going back* recorded only for the *optimal* demonstrations). In both subfigures, red indicates the PSM2 trajectory and blue indicates the PSM1 trajectory. The overlaid trajectories highlight consistent, convergent paths for *optimal* demonstrations versus the more heterogeneous, divergent paths of *recovery* demonstrations.

Subsequently, we assessed the kinematic domain to detect potential artifacts, such as sudden spikes in instrument motion (see Fig. 10). For each demonstration, we calculated the delta in translational distances and angular orientations between consecutive time points, enabling the identification of abrupt trajectory changes. Across individual tissues, the average maximum translational distances recorded were 1.72 mm for PSM1 and 1.60 mm for PSM2. The average maximum angular orientation change was 6.64° for PSM1 (with one notable outlier at 12.32°) and 2.77° for PSM2.

**Fig. 11:**
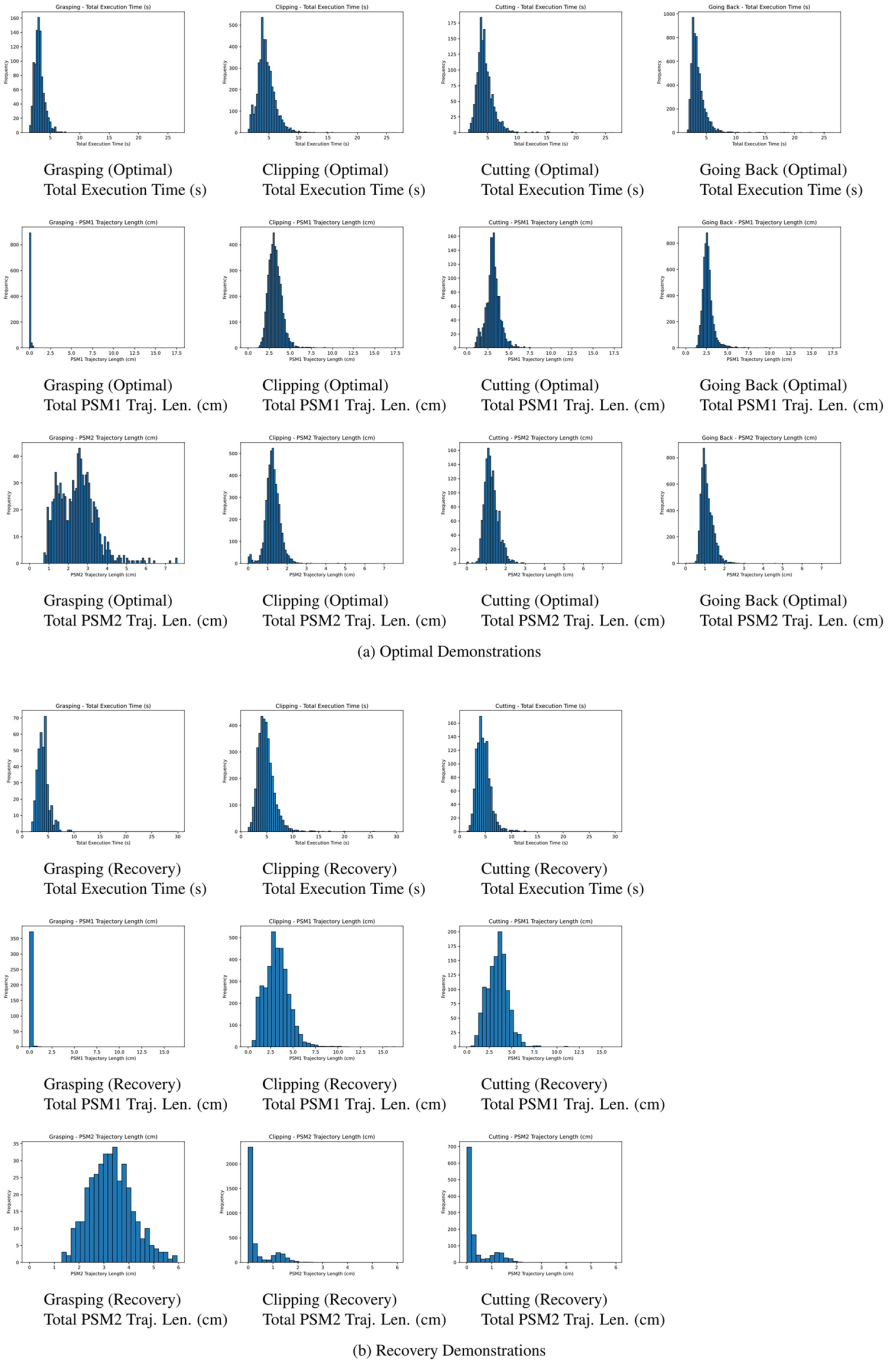
Execution Time & Trajectory Lengths Statistics. Histograms of total execution time, and PSM1/PSM2 trajectory lengths (rows) across tasks—*grasping*, *clipping*, *cutting*, and *going back* (columns)—for *optimal* and *recovery* demonstrations (*going back* only in *optimal*). PSM2 handles grasping; PSM1 performs clipping and cutting.

In a final detailed validation step, we examined outlier cases by selecting the demonstrations with the 20 shortest and longest execution times and cumulative path lengths for both PSM1 and PSM2 (see Fig. 11). These demonstrations underwent a thorough review to confirm their correctness in both modalities and to ensure that the visual recordings correspond to the overall kinematic sequence. Movements observed in the videos were manually compared to their corresponding kinematic trajectories to confirm consistency between both modalities. All reviewed outliers exhibited correct and consistent behavior.

Beyond this subset check, the internal consistency of the dataset is further supported by prior work^16^ using these recordings, in which learned policies successfully reproduced the clipping and cutting phase on eight unseen *ex vivo* tissues, indicating that the visual and kinematic modalities are sufficiently coherent for learning robust phase-level behaviors.

### Validation of Task Labels

Demonstration labels were directly assigned based on their parent surgical task folders. Validation of these labels was performed through the previously described visual inspections, particularly by evaluating the start and end frames and verifying the appropriate folder placement. These steps ensured each demonstration accurately represented its intended task, confirming label correctness implicitly through this broader validation process.

### Limitations of the Dataset

The ImitateCholec dataset has several limitations that should be acknowledged. The dataset was collected using a single surgical robotic system (dVRK Si). Thus, its generalizability to other robotic setups remains uncertain. Although ImitateCholec captures multiple fine-grained tasks involved in clipping and cutting, its restriction to this specific phase of cholecystectomy limits its representation of the full procedural complexity seen in other surgical contexts.

During initial data acquisition, several discrepancies arose in the positioning and orientation of wrist-mounted cameras. Until tissue 4, wrist cameras were positioned closer than the default 3.5 cm distance. For tissue 2, improper mounting of the PSM2 wrist camera caused a 180-degree rotation. Tissues 3 to 9 exhibited variations in PSM1 wrist camera rotation, resulting in altered perspectives. These inconsistencies can be addressed through rectification and augmentation strategies, with detailed transformation procedures and implementation code available in our public repository.

The dataset does not include hardware-level synchronization across sensors. Although all streams were sampled concurrently, the cameras and kinematic sources do not share hardware clocks or trigger signals, and exact multi-view or video-kinematic synchronization cannot be guaranteed.

Despite these limitations, ImitateCholec lays a strong foundation for advancing the development of autonomous robotic systems through imitation learning by providing data for the entire clipping and cutting phase for cholecystectomy.

## Usage Notes

The dataset is published under a Creative Commons Attribution 4.0 International (CC BY 4.0) license, which permits unrestricted use and distribution provided the original work is properly cited.

This dataset is specifically developed to support imitation learning in the context of long-horizon surgical workflows, encompassing entire surgical phases composed of sequential surgical tasks. It can be used to train custom policies via imitation learning to autonomously perform the clipping and cutting phase of cholecystectomy on *ex vivo* porcine tissue. Moreover, the dataset facilitates co-training for novel surgical tasks—a strategy demonstrated to improve task performance and data efficiency in recent imitation learning studies^21,22^. The dataset provides core surgical primitives (grasping, retraction, clipping, cutting) that recur across many procedures, enabling co-trained models to learn transferable behaviors that support generalization beyond the specific phase captured here. Using the action representations proposed in^7,16^, this data can also be used for other dual-arm surgical systems that support Cartesian space control, such as a da Vinci Classic system.

As the dataset is designed for imitation learning, the surgical phases are not captured as continuous recordings but are instead segmented into fine-grained task-specific sequences. To use the dataset for surgical workflow analysis over the entire clipping and cutting phases, neighboring task recordings can be concatenated to synthetically reconstruct full surgical phase recordings. This allows for the simulation of task transitions and the modeling of phase-level surgical workflows. A protocol modification was introduced starting from tissue 9: the retraction of the gallbladder neck was moved from task 2 (clipping first clip left tube) to task 1 (grasping gallbladder). This adjustment aimed to enhance safety and success rates in imitation learning but introduced an inconsistency in the dataset, complicating workflow analysis due to altered task boundaries. To maintain consistency across the dataset, we recommend excluding task 1 from the annotated tasks and start the workflow analysis from task 2.

The dataset also enables the development of surgical tool pose estimation by providing video data and corresponding kinematic recordings of the surgical instruments.

For general clinical use, the inclusion of recovery demonstrations further enables the analysis of failure states, providing valuable insight into error recognition and correction within the surgical workflow. This could support future trainee-guidance systems, for example, in training scenarios (*e.g*., cadaveric setups), before performing the clipping or cutting step by suggesting simple corrective primitives such as moving the tool slightly upward, downward, or toward/away from the surgeon for either instrument, and by notifying the trainee when an erroneous action has been performed.

## Data Availability

The dataset supporting this study is publicly available in the Johns Hopkins Research Data Repository^20^. The repository is open access, requires no user registration, and provides unrestricted access to all data described in this manuscript.

## Code availability

A publicly accessible code repository has been developed to support the utilization of the dataset. It provides comprehensive instructions for loading and preprocessing the dataset, facilitating its integration into a basic training pipeline for the proposed use cases. The repository also provides visualization tools for both visual and kinematic modalities. All scripts are written in Python (evaluated on 3.12.8) and built upon the PyTorch framework (version ≥ 2.0). The code has been tested on Linux platforms. A detailed README file accompanies the repository, offering step-by-step guidance and descriptions of the available components. The full codebase is released under the MIT Open Source license and is available at: https://github.com/phnib/ImitateCholec.
